# Combination of Fenugreek and Quinoa Husk as Sources of Steroidal and Triterpenoid Saponins: Bioactivity of Their Co-Extracts and Hydrolysates

**DOI:** 10.3390/foods13040562

**Published:** 2024-02-12

**Authors:** Emma Cantero-Bahillo, Joaquín Navarro del Hierro, María de las Nieves Siles-Sánchez, Laura Jaime, Susana Santoyo, Diana Martin

**Affiliations:** 1 Sección Departamental de Ciencias de la Alimentación, Facultad de Ciencias, Universidad Autónoma de Madrid, 28049 Madrid, Spain; emma.cantero@uam.es (E.C.-B.); maria.siles@uam.es (M.d.l.N.S.-S.); laura.jaime@uam.es (L.J.); susana.santoyo@uam.es (S.S.); diana.martin@uam.es (D.M.); 2 Departamento de Producción y Caracterización de Nuevos Alimentos, Instituto de Investigación en Ciencias de la Alimentación (CIAL) (CSIC-UAM), 28049 Madrid, Spain; 3 Sección Departamental de Tecnología Alimentaria, Facultad de Veterinaria, Universidad Complutense de Madrid, 28040 Madrid, Spain

**Keywords:** ultrasound-assisted extraction, co-extraction, diosgenin, oleanolic acid, hypocholesterolemic, anti-inflammatory

## Abstract

Saponins, both steroidal and triterpenoid, exhibit distinct bioactivities. However, they are not commonly found together in natural sources; instead, sources tend to be rich in one type or another and mainly in the form of saponins rather than the sapogenin aglycones. Developing co-extracts containing both saponin or sapogenin types would be a strategy to harness their respective bioactivities, yielding multibioactive extracts. Therefore, this study evaluates the bioactivity (hypolipidemic, antioxidant, and anti-inflammatory activities) of co-extracts from fenugreek seeds (steroidal-rich saponins) and quinoa husk (triterpenoid-rich saponins), co-extracted at varying proportions, alongside their respective sapogenin-rich hydrolysates. Pancreatic lipase inhibition increased with fenugreek content in co-extracts, especially in sapogenin-rich variants. The latter substantially interfered with cholesterol bioaccessibility (90% vs. 15% in sapogenin-rich extracts). Saponin-rich co-extracts exhibited reduced cytokine release with increased fenugreek content, while sapogenin-rich counterparts showed greater reductions with higher quinoa husk content. Limited cellular antioxidant activities were observed in all extracts, with improved post-hydrolysis bioactivity. Therefore, simultaneous co-extraction of steroidal and triterpenoid sources, such as fenugreek and quinoa husk, as well as their subsequent hydrolysis, are innovative strategies for obtaining multibioactive natural extracts.

## 1. Introduction

Saponins, one of the main secondary metabolites present in some plants, have been considered antinutrients for many years; although, in recent years, they have awakened interest in the scientific community due to their bioactive potential [1,2]. In general, saponins have been shown to have bioactive properties such as being hypocholesterolemic, anti-inflammatory, and antioxidant, among others [3,4,5]. However, their physicochemical and biological properties vary according to the type of saponin [6]. Thus, they can be classified according to the chemical structure of the sapogenin skeleton into steroidal or triterpenoid saponins. As examples of natural sources, fenugreek and quinoa husk are among the main sources rich in steroidal and triterpenoid saponins, respectively. Specifically, diosgenin has been documented as the main sapogenin in fenugreek, while quinoa husk is known for containing oleanolic acid as its most popular sapogenin, both molecules being widely known for their great bioactive potential. For example, extracts rich in steroidal saponins from fenugreek are noted, both in vitro and in vivo, for their hypolipidemic and antidiabetic activities [7]. On the other hand, quinoa seeds have also been extensively studied in vitro and in vivo for their anti-inflammatory effect, as shown in a recent systematic review in which saponins were studied as one of the main bioactive compounds responsible for this bioactivity [8]. We have also previously demonstrated promising results for steroidal and triterpenoid saponins from fenugreek and quinoa, respectively, in terms of their enzyme inhibitory activities and potential hypocholesterolemic, antioxidant, and prebiotic effects, among others [1,9,10]. Therefore, quinoa husk and fenugreek represent interesting sources for producing bioactive extracts with multiple and different bioactivities depending on the type of saponin, which is of increasing health-related scientific interest.

It is crucial to acknowledge that, typically, natural sources do not contain both steroidal and triterpenoid saponins simultaneously, despite the significant interest in these two types of saponins [11,12]. On the contrary, natural sources tend to be rich in one type of saponin or another, and the respective predominance of triterpenoid or steroidal saponins will depend on the type of source, as for quinoa husk and fenugreek, respectively. Therefore, the combination of different sources rich in each type of saponin may be of interest to produce innovative extracts simultaneously rich in triterpenoid and steroidal saponins not occurring naturally. This approach might allow the production of multibioactive extracts due to the combination of the different bioactivities provided by both types of saponins in one single product. Additionally, thanks to the co-extraction strategy, production costs may be also reduced considerably by reducing the amount of solvent used, the processing time, and the energy required in a single extraction of both sources simultaneously. As an example, some authors have carried out co-extractions to increase the content of bioactive compounds [13]. Other authors have performed co-extractions to enhance a certain bioactivity, such Vázquez et al. [14] who performed the simultaneous extraction of rosemary and spinach leaves to enhance the antioxidant activity. Recently, synergistic effects on the antioxidant activity of marjoram and rosemary co-extracts obtained via pressurized liquid extraction have been demonstrated in all the different proportions studied [15]. However, previous research on the co-extraction of different natural matrices rich in steroidal and triterpenoid saponins in order to obtain extracts enriched in both types of saponins simultaneously has not been found.

Concerning saponins, it is important to remark that the removal of the sugar chains of the molecule of saponins can result in the release of the sapogenin. Sapogenins have shown to have a greater bioactive effect in certain bioactivities than their precursor saponin [1]. However, non-glycosylated sapogenins are rarely found in plant sources. Therefore, the use of hydrolysis strategies to release the sapogenins from saponins has garnered significant attention, as this approach aims to yield sapogenin-rich extracts with enhanced bioactivities with respect to the former saponin-rich extracts [10,16,17,18,19]. In this sense, the combination of the proposed initial step of co-extraction of saponins from different natural sources, followed by the transformation into sapogenin-rich co-extracts, can be approached as a novel strategy to develop innovative bioactive products enriched in free triterpenoid and steroidal molecules simultaneously, that do not occur in food sources naturally. This approach may be an additional strategy to produce multibioactive extracts that combine the different bioactivities provided by both types of sapogenins in one single product but with potentially enhanced bioactivities with respect to the former saponin-rich co-extracts.

The aim of this study was to produce, evaluate, and compare the bioactive potential of saponin-rich and sapogenin-rich co-extracts from quinoa husk (as a source of triterpenoid-like saponins) and fenugreek seeds (as a source of steroid-like saponins). Different extracts with different concentrations of steroid-like and triterpenoid-like saponins were produced by combining different proportions of fenugreek and quinoa husk during the extraction. Then, the hydrolysis of such saponin-rich co-extracts was performed to yield sapogenin-rich co-extracts with different contents of steroid-like or triterpenoid-like aglycones. The potential combination of multibioactivities of the co-extracts and hydrolyzed co-extracts was assessed in terms of their in vitro ability for a large diversity of biological activities that have been already reported for both the individual extracts of fenugreek and quinoa. These are the hypolipidemic activity, which includes pancreatic lipase enzyme inhibition and interference with cholesterol bioaccessibility, as well as their anti-inflammatory and antioxidant effects in cells.

## 2. Materials and Methods

### 2.1. Reagents and Materials

Quinoa husk powder (*Chenopodium quinoa* Willd.) was kindly provided by Algodonera del Sur S.A. (Lebrija, Sevilla, Spain) and seeds of fenugreek (*Trigonella foenum-graecum* L.) were from Plantafarm S.A. (Villanueva del Condado, León, Spain). Phosphatidyl choline from egg yolk, 4-methylumbelliferyl oleate (4-MUO), bile salts, maleic acid, lipase from porcine pancreas, calcium and sodium chloride, pancreatin from porcine pancreas, amano lipase A from *Aspergillus niger*, trizma base, β-sitosterol (≥70%), diosgenin, oleanolic acid, fluorescent marker 2′,7′-dichlorofluorescein diacetate (DCFH-DA), 2,20-azobis(2-methylpropionamidine) dihydrochloride (ABAP), lipopolysaccharide (LPS) from Escherichia coli 055:B5, phorbol 12-myristate 13-acetate (PMA), and 3-(4,5-dimethylthiazol-2-yl)-2,5-diphenyltetrazolium bromide (MTT) were from Sigma-Aldrich Chemie GmbH (Steinheim, Germany). Hederagenin, gitogenin, and sarsasapogenin were acquired from the company Cymit Química S.L. (Barcelona, Spain). Dimethyl sulfoxide (DMSO), ethyl acetate, methanol, and hexane (95% purity) were from Macron (Gliwice, Poland). Hydrochloric acid (2 M) and N,O-bis(trimethylsilyl)trifluoroacetamide (BSTFA) were from Merck KGaA (Darmstadt, Germany). Hanks’ balanced salt solution (HBSS), phosphate-buffered saline (PBS), fetal bovine serum (FBS), 4-(2-hydroxyethyl)-1-piperazineethanesulfonic acid (HEPES), penicillin, non-essential amino acids, L-glutamine, and RPMI 1640 medium were from Gibco (Waltham, MA, USA). Human colorectal adenocarcinoma-derived Caco-2 cell line and human THP-1 monocytes were from ATCC (Manassas, VA, USA). Pro-inflammatory cytokines tumor necrosis factor alpha (TNF-α), interleukin-1β (IL-1β), and IL-6 were from BD Biosciences (Aalst, Belgium). Dulbecco’s Modified Eagle’s Medium (DMEM) was from Corning Inc. (Corning, NY, USA).

### 2.2. Production of Saponin-Rich Co-Extracts

First, hexane (ratio sample to solvent 1:5, *w*/*v*) was used to perform the defatting of each of the samples (fenugreek seed finely ground previously for 1 min in a knife mill, or quinoa husk as powder) for 5 min in an Ultraturrax (IKA, Staufen, Germany) at 11,000 rpm. Then, the mixture was centrifuged at 3396× *g* for 10 min at room temperature, the supernatant was removed, and the precipitate was defatted again following the same procedure. The resulting defatted powder was dried at 50 °C for 20 min to remove any residual hexane.

Initially, the individual extracts of fenugreek (F100) and quinoa husk (Q100) were produced for comparison with the co-extracts. Additionally, these individual extracts allowed us to determine the initial saponin content of such extracts. Then, taking such data into account, the production of three co-extracts was designed to obtain co-extracts that theoretically contained equivalent triterpenoid and steroidal saponins (50% of each type), a predominance of triterpenoid saponins (closer to 70% of total saponins), or a predominance of steroidal saponins (closer to 70% of total saponins). To reach this theoretical composition, the following proportions of defatted fenugreek and quinoa husk were mixed for the extraction: F90Q10 (mixture of fenugreek and quinoa at a ratio of 90:10 (*w*/*w*), respectively, for the predominance of steroid-like saponins), F75Q25 (mixture of fenugreek and quinoa at a ratio of 75:25 (*w*/*w*), respectively, in order to reach around 50% of total saponins as steroid-like and 50% as triterpenoid-like), and F50Q50 (mixture of fenugreek and quinoa at a ratio of 50:50 (*w*/*w*), respectively, for the predominance of triterpenoid-like saponins).

Extractions were performed via ultrasound-assisted extraction, based on Navarro del Hierro et al. [19]. Methanol and water (1:1) (sample to solvent ratio of 1:10, *w*/*v*) was used to extract samples via direct sonication (Branson SFX250 Digital Sonifier, Branson Ultrasonic, Danbury, CT, USA) employing an ultrasonic probe (1/2″ diameter) at 20 kHz and output sonication amplitude of 60% in continuous pulse for 15 min. The mixture was then centrifuged at 2683× *g* for 15 min. The supernatant was collected and evaporated under vacuum to remove methanol. The remaining aqueous phase was freeze-dried (LyoQuest, Telstar, Barcelona, Spain). The whole extraction process was repeated five times for each of the extracts and co-extracts in order to yield five replicates to estimate the extraction yield and saponin content. The replicates were combined after into a single saponin-rich extract or co-extract of each kind (F100, F90Q10, F75Q25, F50Q50, and Q100) for further determination of their bioactivities as well as the production of the hydrolyzed co-extracts. Samples were stored at −20 °C until further use.

### 2.3. Production of Sapogenin-Rich Co-Extracts

Each of the saponin-rich extracts and co-extracts previously obtained were hydrolyzed based on Navarro Del Hierro et al. [10] via microwave-assisted acid hydrolysis. Samples were solubilized in HCl (2 M) at a ratio of sample to acid solution of 1:50 (*w*/*v*), placed into high-pressure vessels and built into the complete segments from an MLS 1200 Mega microwave system (Milestone Srl, Sorisole, Italy). Hydrolysis was performed at 140 °C for 30 min. The theorical power of the instrument was 800 W but the actual power delivered inside the cavity was 752 W, determined indirectly according to Bizzi et al. [20] by measuring the temperature rise in the water after microwave irradiation. To measure the temperature of the samples, an optical fiber temperature sensor was directly inserted into a control high-pressure vessel filled with distilled water. After 30 min, the whole segment was put on ice for 20 min and then the hydrolyzed samples were extracted using ethyl acetate at a ratio of 1:1 (*v*/*v*) via vortex agitation during 1 min. The mixture was centrifuged at 3400× *g* for 5 min. The supernatant was collected, and the precipitate was extracted again under the same conditions using ethyl acetate. Both supernatants were combined and evaporated until dryness. The hydrolysis procedure was performed at least in duplicate, and each of the extracts or co-extracts replicates were combined into a single hydrolyzed extract or co-extract of each kind (HF100, HF90Q10, HF75Q25, HF50Q50, and HQ100) that were stored at −20 °C until the further determination of their bioactivities.

### 2.4. Analysis and Quantification of Saponins and Sapogenins

The quantitative determination of the saponin content in all the extracts and co-extracts was carried out using an HPLC system (LC-2010C 3D Plus System, Shimadzu Corporation, Kyoto, Japan) with diode array detection, based on the method described by Herrera et al. [1]. Quantification of saponins was performed using calibration curves obtained from commercially available saponin standards: protodioscin for the quantification of steroidal saponins from fenugreek and hederacoside C for triterpenoid saponins from quinoa husk. The saponin content was expressed as total saponins (steroidal/triterpenoid) per 100 g of extract.

The quantitative determination of the sapogenin content of the hydrolyzed extracts and co-extracts was performed in an Agilent 7890A GC-MS FID (Agilent Technologies, Santa Clara, CA, USA) via previous derivatization using BSTFA according to Herrera et al. [18]. The quantification of sapogenins was performed using the calibration curves obtained via commercial standards, under the same conditions. Diosgenin, sarsasapogenin and gitogenin were used to quantify fenugreek sapogenins. Oleanolic acid and hederagenin were employed to quantify quinoa sapogenins.

### 2.5. Bioactivity Assessment of the Extracts and Co-Extracts

#### 2.5.1. Pancreatic Lipase Inhibition Assay

The inhibitory activity against the pancreatic lipase enzyme of the extracts and co-extracts was assessed using 4-MUO as a substrate under simulated in vitro intestinal conditions, following the methodology outlined by Herrera et al. [1]. To mimic the intestinal environment, a digestion solution was prepared, comprising 100 mM Trizma-Maleic buffer (pH 7.5), 0.15 M NaCl, and 5.1 mM CaCl_2_, supplemented with bile salts at a concentration of 7.8 mg/mL and lecithin at 3.12 mg/mL. The reaction mixture consisted of 0.5 mL of extract or co-extract solutions in the digestion buffer, at varying concentrations; 0.5 mL of freshly prepared pancreatic lipase at a concentration of 1 mg/mL (prepared by dissolving 10 mg of lipase in 10 mL of digestion buffer, stirring for 10 min, and centrifuging at 2683× *g* for 10 min) was combined with 1 mL of 4-MUO solution at a concentration of 0.1 mM in digestion buffer. The extracts were tested at six different concentrations within the range of 0.06 to 1.5 mg/mL to determine the IC_50_, with each concentration prepared in triplicate. Control samples were also prepared without extracts following the same procedure, and control samples of extracts at different concentrations were prepared without lipase and substrate. Both types of controls were created in triplicate. The samples were shielded from light and incubated in an orbital incubator at 37 °C and 250 rpm for 20 min. Subsequently, three aliquots of 0.150 mL each were transferred to a 96-well plate, and the amount of 4-MUO hydrolyzed using lipase was quantified using a fluorescence microplate reader (Infinite M200, Tecan, Barcelona, Spain), which was set at an excitation wavelength of 350 ± 10 nm and an emission wavelength of 450 nm. The inhibition of pancreatic lipase activity was calculated as follows:

$$Lipase\;Inhibition\left(\%\right)=100-\left[\left(\frac{F_{extract\;sample}-F_{extract\;control}}{F_{control\;sample}}\right)\times 100\right]$$

#### 2.5.2. Hypocholesterolemic Effect via Inhibition of Cholesterol Bioaccessibility In Vitro

The determination of the potential hypocholesterolemic effect of the extracts and co-extracts was carried out based on Navarro del Hierro et al. [21] with modifications. Gastrointestinal digestions of cholesterol were performed in the presence and absence of the experimental samples. The entire process of digestion was performed in tubes of 50 mL capacity. First, a lipid mixture containing 3 mg of lecithin, 8 mg of cholesterol, 80 mg of refined olive oil, and 5 mg of extract was prepared. A positive control (containing 5 mg of β-sitosterol) and a negative control (in the absence of extracts) were also prepared. The final concentration of the extracts in the digestion medium was 1 mg/mL.

For the gastric digestion, 2.2 mL of a gastric solution (150 mM NaCl, 6 mM CaCl_2_, and 0.1 mM HCl) at pH 2.5 and 0.45 mL of a fresh extract of gastric enzymes (16.7 mg/mL of gastric lipase and 29.4 mg/mL of pepsin) were added. This mixture was stirred in an orbital incubator (Titramax 1000 package, Heidolph Instruments, Schwabach, Germany) at 37 °C and 250 rpm for 45 min. After this time, for the intestinal digestion, 1.9 mL of a solution simulating a biliary secretion (8.3 mg of lecithin/mL and 20.8 mg bile salts/mL in 5 mL of trizma-maleate buffer 100 mM pH 7.5, plus 0.25 mL of 350 mM CaCl_2_ solution and 0.75 mL of 3.25 M NaCl solution, stirred for 10 min) was added, and the mixture was stirred for 2 min at 37 °C and 190 rpm. The intestinal digestion was initiated by the addition of 0.45 mL of a fresh pancreatic extract at 166.7 mg/mL in trizma-base buffer, which had been previously stirred for 10 min and centrifugated for 15 min at 2683× *g*. The digestion was performed at 190 rpm for 60 min, at least in duplicate for each sample.

To determine the bioaccessibility of cholesterol, the digestion medium underwent centrifugation for 40 min at 2683× *g*. Following centrifugation, 3 mL of the micellar phase, containing solubilized cholesterol, was collected and subjected to extraction using ethyl acetate at a 1:1 (*v*/*v*) ratio. The mixture was vortexed for 5 min and then centrifuged at 2054× *g* for 5 min. The upper phase was collected and directly analyzed using an LC-2030C 3D Plus system (Shimadzu, Kyoto, Japan) for cholesterol quantification. The separation of compounds was carried out on an ACE 3 C18-AR column (150 mm × 4.6 mm, 3 µm particle size) with protection from a guard column (Avantor, Radnor, PA, USA). Chromatographic analysis followed the method described by Kolarič and Šimko [22], utilizing an isocratic flow of methanol with 0.05% water as the mobile phase. The flow rate remained constant at 1.2 mL/min, and the column temperature was maintained at 35 °C. An injection volume of 10 µL was used. UV–Visible spectra were recorded from 190 to 800 nm, and chromatograms were registered at 205 nm. The quantification of cholesterol was carried out using a calibration curve generated from its corresponding commercial standard.

#### 2.5.3. Cellular Antioxidant Activity

After confirmation of the non-cytotoxic effect of the extracts and co-extracts in Caco-2 cells using a MTT test [23], the cellular antioxidant activity (CAA) was tested following the method described by Wolfe and Liu [24], with modifications. Caco-2 cells were plated at a density of 2 × 10^5^ cells/mL in 96-well plates. The medium was removed after 48 h and PBS was used to wash the cells. Subsequently, cells were incubated with 25 μM of fluorescent marker DCFH-DA and extracts or co-extracts in subtoxic concentrations (100–1000 µg/mL in DMSO). The medium was removed after 1 h, cells were washed 3 times using PBS, and the free radical initiator ABAP in HBSS (600 μM) was added (600 μL) to each well. Fluorescence readings (Plate Reader Cytation 5, Biotek Instruments Inc., Winooski, VT, USA) were taken every 5 min for 1 h at 37 °C. Excitation/emission wavelengths were, for 13 cycles, 485/538 nm. The reduction in fluorescence was calculated by employing the following equation:

$$Inhibition\left(\%\right)=\left(1-\frac{AUC\;sample}{AUC\;blank}\right)\times 100.$$

The IC_50_ value was determined as 50% of inhibition.

#### 2.5.4. Anti-Inflammatory Activity Assay

Human THP-1 monocytes (5 × 10^5^ cells/mL) were seeded in 24-well plates. The culture medium consisted of RPMI 1640 supplemented with 10% FBS, 100 mg/mL streptomycin, 100 U/mL penicillin, and 2 mM L-glutamine in 95% humidified air containing 5% CO_2_ at 37 °C. Cells were maintained for 48 h using 100 ng/mL of PMA to induce the differentiation of monocytes to macrophages (THP-1/M cells). The viability of THP-1/M cells was tested using the MTT assay [23] in presence of extracts and co-extracts (40 µg/mL and 10 µg/mL in DMSO for the saponin-rich and sapogenin-rich extracts and co-extracts, respectively). After differentiation, non-supplemented RPMI was used to wash THP-1/M cells before incubation for 24 h with 0.05 μg/mL of LPS from *E. coli* O55:B5 in the presence of extracts. Supernatants were collected and frozen at −20 °C. The release of TNF-α, IL-1β, and IL-6 in the supernatants was measured using ELISA kits (BD Biosciences, Aalst, Belgium), according to manufacturer’s instructions. The quantification was performed using an Infinite M200 multiscanner autoreader (Tecan, Barcelona, Spain) at 450 nm with substrate correction at 570 nm. The results are expressed as the mean of three determinations ± standard deviation.

### 2.6. Statistical Analysis

The SPSS 26.0 statistical package (SPSS Inc., Chicago, IL, USA) was used to perform statistical analyses by means of the general linear model procedure of the one-way analysis of variance. Differences were considered significant at *p* ≤ 0.05. In order to establish significant differences, post hoc Tukey’s tests were performed. Pearson correlation tests were conducted for further analyses.

## 3. Results and Discussion

### 3.1. Saponin and Sapogenin Content of the Extracts and Co-Extracts

In the present study, fenugreek and quinoa husk extracts and co-extracts were produced in different proportions (F100, F90Q10, F75Q25, F50Q50, and Q100). Ratios were selected based on the desired final proportion of steroid-like and triterpenoid-like saponins in the co-extracts (either the predominance of steroidal over triterpenoid, F90Q10, similar amounts of steroidal and triterpenoid, F75Q25, or the predominance of triterpenoid over steroidal, F50Q50). Then, each of the extracts and co-extracts were subjected to hydrolysis to obtain the corresponding sapogenin-rich extracts (HF100, HF90Q10, HF75Q25, HF50Q50, and HQ100).

The total saponin and sapogenin contents of each of the extracts and co-extracts, as well as the final obtained ratio of steroid-like or triterpenoid-like saponins or sapogenins are shown in Table 1. As expected, the extracts produced from fenugreek (F100) or quinoa husk (Q100) contained a single type of saponin (steroidal or triterpenoid, respectively). However, under the same conditions of extraction, quinoa husk yielded extracts with a much higher concentration of saponins compared to fenugreek. When co-extracted, the reached content of steroid-like plus triterpenoid-like saponins in the extracts was very similar to the theoretically estimated content, with expected divergences due to the impact of the extraction process itself on the final achieved concentration of the targeted compounds.

After hydrolysis, the main sapogenins from fenugreek and quinoa husk were quantified. Similar to the saponin-rich extracts, the concentration of sapogenins was three times superior for HQ100 compared to HF100 (Table 1). For the hydrolyzed co-extracts, the production of steroid plus triterpenoid sapogenin-rich extracts was reached, but the proportion of triterpenoid-like sapogenins was higher in all cases by more than 60%. This could be related to the hydrolysis yield and the conversion of saponins to sapogenins, which is higher in the case of quinoa husk than fenugreek, as documented by Herrera et al. [18]. Additionally, the simultaneous release, together with the unavoidable partial degradation of sapogenins that takes place during the hydrolysis process, which is sapogenin-type dependent [10], also makes it difficult to reach a precise final content of each sapogenin when co-hydrolyzed after co-extraction.

### 3.2. Inhibition Activity against Pancreatic Lipase of Extracts and Co-Extracts

Pancreatic lipase is a digestive enzyme involved in the digestion of dietary triglycerides for their absorption and utilization by the body. The inhibition of the enzyme is considered to be a simple strategy to prevent the increase of lipids and cholesterol in the blood, which has been extensively associated with an increased risk of chronic diseases. We have documented previously this activity for fenugreek and quinoa extracts [1,21]. Therefore, taking into account the positive bioactivity of each of the extracts individually, the aim was to test a potential synergistic or additive effect when both sources were co-extracted together into a single extract in which triterpenoid and steroidal saponins co-exist, as well as together with other different co-extracted compounds that could also contribute to the overall lipase-inhibitory bioactivity of the co-extracts.

**Figure 1:**
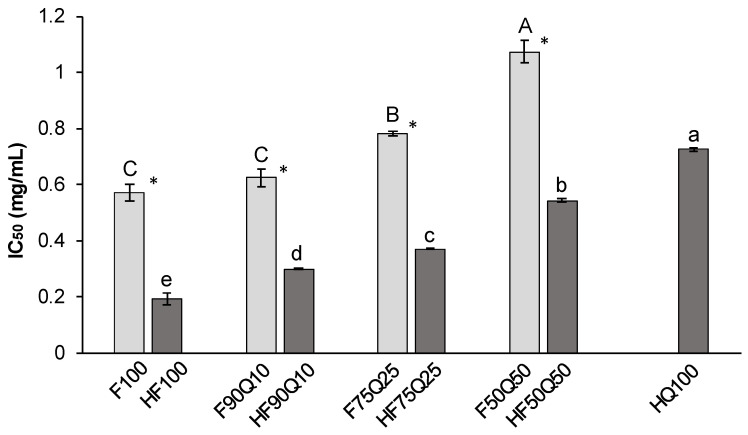
IC_50_ values (mg/mL) of saponin-rich and sapogenin-rich extracts and co-extracts from fenugreek seeds and quinoa husk against pancreatic lipase. (*) Values of IC_50_ for saponin-rich and sapogenin-rich extracts and co-extracts within each sample are significantly different (*p* ≤ 0.05). Mean values with different letters (^A–C^, saponin-rich extracts and co-extracts; ^a–e^, sapogenin-rich extracts and co-extracts) are significantly different (*p* ≤ 0.05).

Focusing first on the saponin-rich extracts and co-extracts, most of them showed inhibitory activity against the pancreatic lipase in a dose-dependent manner, which allowed us to estimate the IC_50_ values (Figure 1). The inhibitory activity varied among the co-extracts (*p* = 0.001) and was affected by the predominant type of plant (fenugreek/quinoa). Thus, the inhibitory capacity of the co-extracts decreased as the proportion of quinoa saponins increased in them. Therefore, F90Q10 was the co-extract with the highest inhibitory capacity (IC_50_ = 0.63 ± 0.03 mg/mL), not being different than F100 (IC_50_ = 0.57 ± 0.03 mg/mL). On the contrary, the extracts consisting of quinoa husk only (Q100) showed a poor ability to inhibit the pancreatic lipase (IC_50_ > 1.5 mg/mL, not represented in Figure 1). However, the co-extract F50Q50, which mostly consisted of quinoa saponins (Table 1), showed a non-negligible effect towards the inhibition of the enzyme (IC_50_ = 1.08 ± 0.04 mg/mL), which would be likely due to the co-extraction of fenugreek saponins. Therefore, the presence of a small proportion of fenugreek clearly confers a great enhancement in the inhibitory capacity of a previously non-bioactive Q100 extract. In fact, a significant negative correlation was found between the content of steroidal saponins and the IC_50_ value (r = −0.988; *p* < 0.001), which means a stronger lipase inhibitory activity as the content of steroidal saponins increases in the extracts. Nevertheless, the contribution of other co-extracted compounds from fenugreek to this bioactivity should not be ruled out.

In the case of the sapogenin-rich extracts and co-extracts, all of them also possessed inhibitory activity against the pancreatic lipase in a dose-dependent manner, and the IC_50_ values were estimated accordingly (Figure 1). In general terms, this inhibitory activity was significantly stronger than that of the saponin-rich extracts and co-extracts (*p* = 0.003). Similar to what occurred for the saponin-rich extracts, the inhibitory activity increased along with the fenugreek content in the co-extracts. Thus, the strongest IC_50_ value was observed for HF100 (IC_50_ = 0.19 ± 0.02 mg/mL), followed by HF90Q10 which showed the lowest IC_50_ value among the three co-extracts (0.30 ± 0.00 mg/mL). However, it seems that this outcome was not followed by a synergistic effect between the compounds from quinoa husk and from fenugreek contained in the co-extracts; hence, the lipase-inhibitory potential of the hydrolyzed extracts relied mostly on the combined fenugreek proportion.

A remarkable result was that, although HQ100 was the hydrolyzed extract with the worst inhibitory activity (0.73 ± 0.00 mg/mL), a great improvement in its inhibitory capacity was observed after the hydrolysis process compared to the non-hydrolyzed Q100 extract.

The literature comparing the bioactivity of saponin-rich extracts and their hydrolysates is scarce, and studies evaluating the lipase-inhibitory effect of co-extracts from natural sources are inexistent. We already showed a higher lipase inhibitory capacity for fenugreek extract (IC_50_ = 0.7 mg/mL) compared to the quinoa extract (IC_50_ = 2 mg/mL), whose values were very similar to those of the present study [1]. We also performed the hydrolysis in acidic conditions of extracts rich in saponins obtained from these two seeds using conventional procedure, observing a greater inhibitory effect for the hydrolyzed extract of quinoa compared to the non-hydrolyzed one [21], in agreement with the current study, but performed using microwave-assisted acid hydrolysis in this case. In the current study, the saponin contents found in the fenugreek and quinoa extracts were notably higher compared to the initial saponin purity of the saponin-rich extracts in our previous study. Furthermore, in the previous case, whole quinoa seeds were used, while quinoa husk has been used in the current study as a richer source of triterpenoid saponins instead. Therefore, the present study confirms the suitability of the hydrolysis process to produce extracts with enhanced bioactivity and provides evidence for the first time that this strategy is also useful in case of co-extracts.

### 3.3. Effect of Extracts and Co-Extracts on the Bioaccessibility of Cholesterol

The potential cholesterol-lowering effect of all the extracts and co-extracts was examined using an in vitro model of gastrointestinal digestion. Although the mechanism whereby saponin-rich extracts interfere with the bioaccessibility of cholesterol is still uncertain, some possible explanations have been proposed—for example, the precipitation of cholesterol via a decrease in micelle formation due to the precipitation of bile salts, which results in a reduced solubilization capacity of the digestion mixture; the formation of insoluble cholesterol-saponin complexes due to their strong affinity to the reaction mixture; and the displacement of cholesterol from micelles via components of the extracts, leading to an increase in cholesterol in the aqueous medium and, consequently, its precipitation [25]. Here, we assessed the ability of the extracts and co-extracts to interfere with the micellar solubility of cholesterol, or bioaccessibility. In addition, digestions in absence of extracts (negative control) and in presence of β-sitosterol (positive controls) were performed.

**Figure 2:**
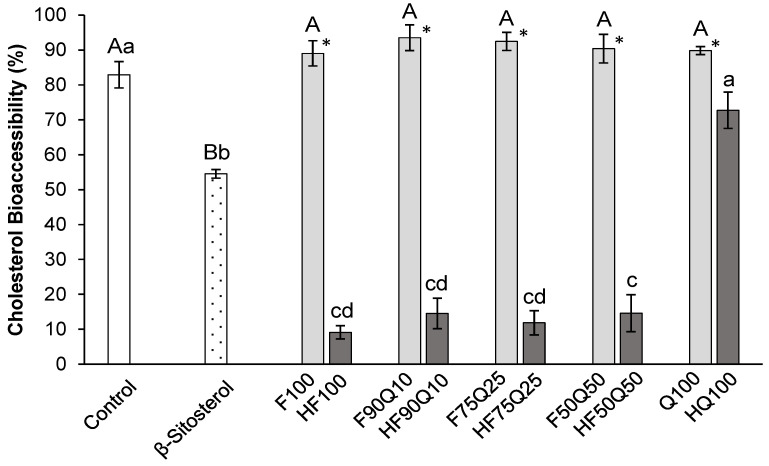
Bioaccessibility of cholesterol (%) after gastrointestinal digestion of cholesterol in absence (control) and presence of β-sitosterol, saponin-rich and sapogenin-rich extracts, and co-extracts of fenugreek seeds and quinoa husk. (*) Values of cholesterol bioaccessibility for saponin-rich and sapogenin-rich extracts within each sample are significantly different (*p* ≤ 0.05). ^A,B^ Different letters between saponin-rich extracts, β-sitosterol, and control are significantly different (*p* ≤ 0.05). ^a–d^ Different letters between sapogenin-rich extracts, β-sitosterol, and control are significantly different (*p* ≤ 0.05).

The bioaccessibility of cholesterol at the end of the digestion for all the assayed samples is shown in Figure 2. First, none of the saponin-rich extracts and co-extracts caused a significant effect on the reduction of the bioaccessibility of cholesterol, which remained the same as the control digestion. This similar outcome was recently documented for other extracts from fenugreek and quinoa, although with lower saponin contents [21]. The fact that the extracts and co-extracts produced in the present study did not reduce the bioaccessibility of cholesterol, showing an even higher saponin content than our previous study, confirms that extracts rich in saponins from quinoa and fenugreek, or their combinations, do not interfere with the micellization of cholesterol and further solubility in the intestinal tract. Although we have not observed a hypocholesterolemic effect for these saponin-rich extracts, other authors have shown a decrease in the solubility of cholesterol in the digestion medium in the presence of saponin-rich extracts from other plant sources such as *Quillaja saponaria* [25]. Therefore, taking into account the results achieved, it seems that the potential of saponin-rich extracts to impact the bioaccessibility of cholesterol cannot be generalized. Additionally, in the case of complex extract, the contribution of other co-extracted compounds to enhancing the bioaccessibility of cholesterol may not be ruled out, as any residual lipid in the co-extracts might be [26].

However, contrary to the saponin-rich extracts, the hydrolysis of all co-extracts and HF100 strongly affected their ability to interfere with the solubility of cholesterol and further bioaccessibility, which was around 14% in general and as low as 9% for HF100. These values imply an approximate 85% reduction in the bioaccessibility of cholesterol, which are very promising results considering that they were significantly lower (*p* < 0.001) than the bioaccessibility of cholesterol in the presence of the positive control β-sitosterol (54.5 ± 1.2%). In the case of HQ100, an improved effect was also noted in contrast to the non-hydrolyzed Q100 extract, but such an improvement was not high enough to differ from the control digestion.

Thus, it can be concluded that fenugreek was responsible for the hypocholesterolemic activity of the hydrolyzed co-extracts, and that same magnitude of effect was observed regardless of the fenugreek proportion. In fact, it is interesting to remark that HF50Q50 contained as much as nearly 36% triterpenoid sapogenins from quinoa and 4% steroidal sapogenins from fenugreek, but the effect was still as strong as the one exhibited by HF100, which contained 16% steroidal sapogenins only. Therefore, together with the action of the steroidal sapogenins, the contribution to the bioactivity of other compounds of fenugreek different to sapogenins should not be ruled out.

We previously showed a hypocholesterolemic effect of a hydrolyzed quinoa extract at both 5 and 10 mg/mL [21], which means a higher concentration than the one used in the present study at 1 mg/mL. We previously noted that a hydrolyzed fenugreek extract had an impact on the bioaccessibility of cholesterol, resulting in a 59% reduction at the highest concentration, whereas in the present study the reduction was much higher at a concentration 10 times lower. These differences are difficult to explain, but they may be due to the origin of the raw material or the different hydrolysis methods. In any case, the current study confirms that a small amount of fenugreek is enough for the extract or co-extract to exhibit a hypocholesterolemic effect and that the hydrolysis process of extracts rich in saponins from quinoa husk and fenugreek is an interesting strategy that allows for the obtention of extracts with this bioactivity. However, despite the concurrent rise in quinoa husk compounds within the hydrolyzed extracts while fenugreek compounds decrease, there appears to be no negative impact on the bioactivity of fenugreek. This suggests that a potential synergistic effect between the compounds of quinoa and fenugreek cannot be dismissed.

### 3.4. Anti-Inflammatory Effect of Extracts and Co-Extracts

TNF-α, IL-1β, and IL-6 are inflammatory-mediating cytokines produced by monocytes/macrophages and are known to have an important role in the pathogenesis of many inflammatory diseases, including those related to metabolic syndrome [27].

First, the assay performed to evaluate the cytotoxicity of the extracts indicated that 40 µg/mL of the saponin-rich and 10 µg/mL of the sapogenin-rich extracts and co-extracts did not compromise THP-1/M cells (data not shown). After confirming the lack of cytotoxic effect, the secretion of inflammatory-mediating cytokines was assessed.

**Figure 3:**
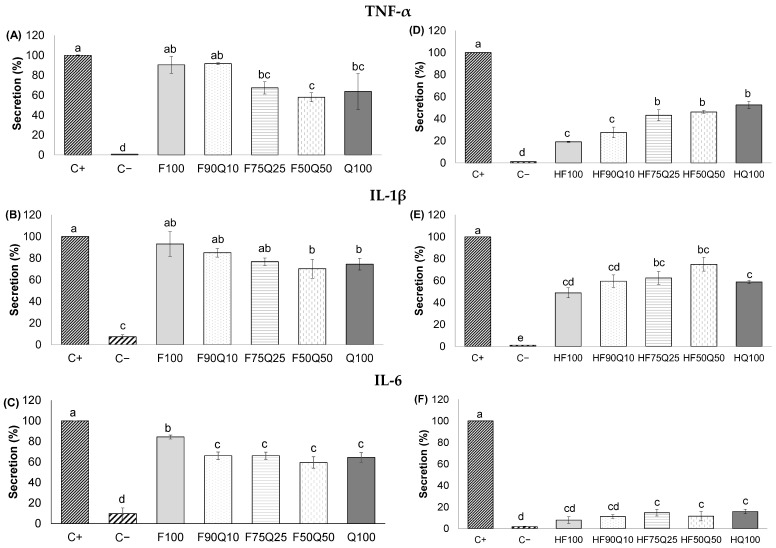
Levels of TNF-α (**A**,**D**), IL-1β (**B**,**E**), and IL-6 (**C**,**F**) secreted by THP-1 macrophages activated with LPS in presence of 40 µg/mL of saponin-rich extracts and co-extracts (**A**–**C**) and 10 µg/mL of sapogenin-rich extracts and co-extracts from fenugreek seeds and quinoa husk (**D**–**F**). Positive control (cells stimulated using LPS in absence of extract), negative control (cells in contact with RPMI medium only). ^a–e^ Different letters indicate statistical differences among samples (*p* ≤ 0.05).

As shown in Figure 3, cells treated with LPS (positive control) exhibited a notable rise in the secretion of TNF-α, IL-6, and IL-1β following a 24 h incubation, in contrast to non-stimulated cells (negative control). The activation of THP-1/M using LPS in the presence of saponin-rich extracts and co-extracts (Figure 3A–C) resulted in reduce secretion overall, particularly as the proportion of quinoa husk increased in the co-extracts, compared to the secretion levels observed in positive controls. Thus, a decrease in TNF-α secretion (Figure 3A) was observed for the co-extracts F75Q25 and F50Q50 compared to the positive control, apparently reaching a plateau of secretion with Q100 (64%). Regarding IL-1β (Figure 3B), a trend to a lower secretion was also observed along with the increase in quinoa husk in the co-extracts F50Q50 and Q100 being capable of significantly lowering the secretion of IL-1β compared to the positive control. For IL-6 (Figure 3C), all co-extracts and Q100 were capable of lowering the secretion of this cytokine in a similar way (60–66%), all of them being significantly lower than F100 (*p* < 0.001). Therefore, the current results clearly showed a relevant anti-inflammatory activity of quinoa husk extracts that is worsened when co-extracted with fenugreek. Yao et al. [28] also observed a dose–response effect for quinoa saponin-rich extracts obtained using different solvent ratios, showing a greater effect on IL-6 than TNF-α. However, in our study, the results were very similar for both cytokines.

Regarding the sapogenin-rich extracts and co-extracts, in general terms, their anti-inflammatory effect was improved after the treatment of hydrolysis (Figure 3D–F). However, in the case of TNF-α (Figure 3D), and contrary to what occurred for the saponin-rich extracts, the incorporation of quinoa husk during the co-extraction reduced the ability of the co-extracts to inhibit the secretion of this cytokine, as HF100 caused a 19% secretion, whereas HQ100 caused a 53% secretion. A similar pattern was observed for IL-1β (Figure 3E), although HQ100 exhibited a stronger inhibitory effect than HF50Q50. In the case of the cytokine IL-6 (Figure 3F), all extracts and co-extracts similarly demonstrated a very strong reduction (around 80%) compared to the positive control. Therefore, in the case of the hydrolyzed co-extracts, and contrary to that observed for saponin-rich extracts, it seems that those compounds released from fenugreek were, in general, more active compared to those from the hydrolyzed quinoa husk extracts.

The information about previous studies dealing with hydrolyzed saponins and their anti-inflammatory activity is scarce. Nevertheless, the anti-inflammatory potential of steroidal sapogenins has been extensively documented. Diosgenin, the main sapogenin found in fenugreek, has been demonstrated to decrease the production of pro-inflammatory mediators in different macrophage cell lines, which has been related to the down-regulation of phosphorylation of transcription factors associated with these cytokines [29]. Yu et al. [30] also observed a decrease in plasma inflammatory cytokines in mice supplemented with 80 mg of sarsasapogenin per kg. Concerning triterpenoid sapogenins, the anti-inflammatory effect of the main sapogenins present in quinoa has also been documented both in vitro and in vivo [31]. Choi et al. [32] also observed higher anti-inflammatory activity from oleanolic acid and hederagenin obtained after the hydrolysis of *Akebia kinata* triterpenoid saponins. Therefore, it is likely that the anti-inflammatory activity of the hydrolyzed extracts and co-extracts in the present study is partially due to sapogenins, among many other compounds contained in the extracts.

### 3.5. Antioxidant Cellular Effect of Co-Extracts

The antioxidant effects of all extracts and co-extracts in Caco-2 cells was performed and the results are shown in Table 2. The cell-based antioxidant assay can be considered a more biologically relevant method than the chemical methods mostly employed because it takes into account some aspects of the metabolism and the uptake of antioxidant compounds in cells [24].

In the case of the saponin-rich extracts and co-extracts, none of them showed antioxidant effect at the concentrations assayed (IC_50_ > 1000 µg/mL) (Table 2). Similarly, Xu and Chang [33] did not observe either a positive correlation between the saponin content and its antioxidant activity in human cancer cell lines. In the specific case of fenugreek and quinoa saponins, previous studies about the cellular antioxidant activity have not been reported. In vitro antioxidant methods have been widely studied such as the β-carotene-linoleate model system and other radicals assays, although most studies have concluded that saponins are not characterized by a potent antioxidant activity [34]. Therefore, our results confirm that the cellular antioxidant activity of saponin-rich extracts and even co-extracts from fenugreek and quinoa husk is inexistent, which agrees with previous non-cellular in vitro studies. This outcome also demonstrates that the extraction and co-extraction of these two natural sources did not favor either of the extraction of other potential compounds with antioxidant ability.

Nevertheless, the hydrolysis of the saponin-rich extracts improved the antioxidant activity of all the sapogenin-rich extracts and co-extracts (Table 2). Similar to what we observed for previous bioactivities in the present study, the higher the fenugreek content in the hydrolyzed co-extracts, the greater the antioxidant effect was in general. In fact, a significant negative correlation (r = −0.750, *p* = 0.005) was found between the IC_50_ value and the steroidal sapogenin content among HF100, HF90Q10, HF75Q25, and HF50Q50, which means that the presence of sapogenins from fenugreek was related to a stronger antioxidant activity. Nevertheless, this correlation did not mean that other compounds of the extracts could not be also involved in such moderated antioxidant activity of the hydrolyzed extracts. Similarly, but using other in vitro techniques, Dixit et al. [35] observed a certain antioxidant capacity in different sapogenin-rich extracts of germinated fenugreek, although a lack of effect was observed when using isolated diosgenin. For triterpenoid sapogenins, oleanolic acid showed little or no ability to inhibit the DPPH radical compared to reference antioxidants such as Trolox, vitamin C, N-acetylcysteine, or butylated hydroxytoluene [36].

Thus, this study reaffirms the value of the hydrolysis process in yielding antioxidant extracts from fenugreek and quinoa husk, which lack bioactivity in their unprocessed state. Additionally, it demonstrates the efficacy of this strategy for co-extracts derived from both sources.

## 4. Conclusions

The co-extraction of quinoa husk and fenugreek seeds at different ratios and their respective hydrolysates results in multibioactive extracts rich in triterpenoid-like and steroid-like saponins or sapogenins whose bioactive potential is favored by the predominance of one plant or another. Thus, when co-extracted together, the pancreatic lipase-inhibitory activities are provided by fenugreek, while anti-inflammatory activity is mainly provided by quinoa husk. Nevertheless, such anti-inflammatory and pancreatic lipase-inhibitory activities for all extracts and co-extracts can even be improved by strategies of hydrolysis of the saponin-rich extracts. Additionally, this transformation of the extracts allows us to obtain extracts with bioactivities not being exhibited by the initial saponin-rich extracts, such as the ability to reduce the bioaccessibility of cholesterol and the antioxidant activity. The presence of fenugreek improves the bioactivity of all hydrolyzed co-extracts.

Thus, simultaneous extraction from different saponin-rich sources such as fenugreek (steroidal-like saponins) and quinoa husk (triterpenoid-like saponins) is shown as an interesting strategy to obtain multibioactive extracts that combine the multiple bioactivities from fenugreek with the great anti-inflammatory activity from quinoa husk. Additionally, in this context of co-extraction strategies, it is possible to modulate a set of desired bioactivities, to enhance the one that is sought as predominant, and to even reveal novel bioactivities by adjusting the proportion of plant material during the co-extraction and/or by applying post-extraction transformation processes, such as acid hydrolysis.

Due to the lack of previous studies focusing on the bioactivity of co-extracts rich in both types of saponins and sapogenins, further studies dealing with other bioactivities and/or sources of triterpenoid-like and steroid-like saponins might be relevant, as well as studies based on the safety, stability, and formulation of co-extracts, in order to augment the possibilities of this innovative extraction model to produce multibioactive extracts. In addition, other extraction and hydrolysis methodologies different to UAE and microwaves, respectively, might be relevant to explore in order to elucidate the bioactivity of co-extracts as affected by the technique used. Finally, further studies may be required to describe the co-existence of other phytochemical compounds different to saponins found in fenugreek and quinoa husk during their co-extraction, which might be involved in the bioactivities described, providing additional value to these novel extracts.

## Figures and Tables

**Table 1:** Total saponin and sapogenin content (g/100 g) of extracts and co-extracts from fenugreek seeds and quinoa husk and proportion (%) of each type (steroidal or triterpenoid) of saponin or sapogenin with respect to the total content.

Sample	Total Saponin Content (g/100 g)	Proportion (%)	
		Steroidal	Triterpenoid
F100	39.9 ± 1.1 ^a^	100	-
F90Q10	49.7 ± 0.5 ^b^	69.8	30.2
F75Q25	53.5 ± 3.7 ^b^	54.6	45.4
F50Q50	61.9 ± 3.6 ^c^	26.2	73.8
Q100	71.9 ± 4.1 ^d^	-	100
Sample	Total sapogenin content (g/100 g)		
HF100	16.4 ± 3.1 ^A^	100	-
HF90Q10	27.1 ± 1.8 ^AB^	31.9	68.1
HF75Q25	23.4 ± 3.7 ^A^	16.7	83.3
HF50Q50	39.3 ± 3.2 ^BC^	9.5	90.5
HQ100	52.3 ± 6.7 ^C^	-	100

Mean values with different letters (^a–d^, saponin-rich extracts and co-extracts; ^A–C^, sapogenin-rich extracts and co-extracts) are significantly different (*p* ≤ 0.05).

**Table 2:** Cellular antioxidant activity of saponin-rich and sapogenin-rich extracts and co-extracts from fenugreek seeds and quinoa husk expressed as *p* values (μg/mL).

Saponin-Rich Extracts and Co-Extracts	IC_50_ (μg/mL)
F100	>1000
F90Q10	>1000
F75Q25	>1000
F50Q50	>1000
Q100	>1000
Sapogenin-rich extracts and co-extracts	
HF100	234.2 ± 34.4 ^d^
HF90Q10	226.2 ± 21.7 ^d^
HF75Q25	393.3 ± 23.3 ^c^
HF50Q50	331.7 ± 12.1 ^b^
HQ100	505.7 ± 31.5 ^a^

^a–d^ Different letters are significantly different (*p* ≤ 0.05).

## Data Availability

The data used to support the findings of this study can be made available by the corresponding author upon request.
